# The cephalic vein catheterization: maybe yes, but when there is no alternative

**DOI:** 10.1186/s13054-023-04321-z

**Published:** 2023-01-18

**Authors:** Ryszard Gawda, Tomasz Czarnik

**Affiliations:** Department of Anesthesiology and Intensive Care, Institute of Medical Sciences, University of Opole, Al. Witosa 26, 45-401 Opole, Poland

Dear Editor,

We read with interest the paper entitled ‘Central venous catheterization: the cephalic vein access’ published in Critical Care [1]. The authors describe a patient who needed central venous cannula exchange for vasopressor administration and invasive hemodynamic monitoring. Therefore, they performed a cephalic vein catheterization.

The presented case report is surprising for a number of reasons. First, the patient already had a central venous cannula inserted via supraclavicular route, so the justification given for the catheter exchange is unclear. Second, the right cephalic vein, chosen for the operation, is 0.28 cm in diameter. As we know, inserted vascular catheters should not exceed 45% of the cross-sectional area of the cannulated vein due to the high risk of thrombotic complications [2]. This means that catheter diameter the authors used should not have exceeded 0.12 cm. However, the inserted catheter shown in Fig. 1 is much far larger [1]. Unfortunately, the authors did not mention this important issue. Third, the readers are not informed why other potentially accessible central veins were not chosen for catheterization (right internal jugular vein or left axillary vein). This is confusing since the right axillary vein presented on the ultrasound image (Fig. 1) seems as if it would have been very convenient for ultrasound-guided catheterization [1]. Four, the cannulated patient was defined as ‘obese,’ and the authors recommend this technique for consideration in morbidly obese patients. However, the body mass index in this case was 28, so the patient should be classified as ‘overweight’ [3].

In our opinion, the case described a unique rescue procedure rather than a reasonable approach for central vein catheterization. Based on this paper, this technique should be perceived strictly as a temporary solution which can be applied only if other central veins are inaccessible.

## Data Availability

Not applicable.
